# Lassa Virus Targeting of Anterior Uvea and Endothelium of Cornea and Conjunctiva in Eye of Guinea Pig Model

**DOI:** 10.3201/eid2505.181254

**Published:** 2019-05

**Authors:** Joy M. Gary, Stephen R. Welch, Jana M. Ritter, JoAnn Coleman-McCray, Thanhthao Huynh, Markus H. Kainulainen, Brigid C. Bollweg, Vaunita Parihar, Stuart T. Nichol, Sherif R. Zaki, Christina F. Spiropoulou, Jessica R. Spengler

**Affiliations:** Centers for Disease Control and Prevention, Atlanta, Georgia, USA

**Keywords:** Lassa fever, Lassa virus, viral hemorrhagic fever, ocular, eye, anterior uvea, endothelial cells, guinea pig, viruses, zoonoses

## Abstract

Lassa virus (LASV), a hemorrhagic fever virus endemic to West Africa, causes conjunctivitis in patients with acute disease. To examine ocular manifestations of LASV, we histologically examined eyes from infected guinea pigs. In fatal disease, LASV immunostaining was most prominent in the anterior uvea, especially in the filtration angle, ciliary body, and iris and in and around vessels in the bulbar conjunctiva and peripheral cornea, where it co-localized with an endothelial marker (platelet endothelial cell adhesion molecule). Antigen was primarily associated with infiltration of T-lymphocytes around vessels in the anterior uvea and with new vessel formation at the peripheral cornea. In animals that exhibited clinical signs but survived infection, eyes had little to no inflammation and no LASV immunostaining 6 weeks after infection. Overall, in this model, LASV antigen was restricted to the anterior uvea and was associated with mild chronic inflammation in animals with severe disease but was not detected in survivors.

Lassa virus (LASV) is the etiologic agent of Lassa fever (LF), a viral hemorrhagic fever endemic to West Africa. Incidence of LF in areas to which it is endemic is ≈100,000–300,000 cases annually, of which ≈5,000 are fatal (*1*). After an incubation period of 7–21 days (*2*,*3*), disease onset is gradual and includes fever, weakness, myositis, and ulcerative pharyngitis that may progress to myocarditis, pneumonitis and pleuritis, and encephalopathy and hemorrhage (*2*). The most well-documented sequela of LF is hearing loss (*4*–*8*).

Ocular involvement in acute LF includes conjunctivitis and conjunctival edema (*9*). In addition, transient blindness has been described in humans recovering from LASV infection (*3*,*10*). The extent of viral presence in the eye, the ocular structures targeted by LASV, and the clinical implications of ocular infection are unknown.

In viral hemorrhagic fever disease, ocular manifestations are not limited to LF and are well described for infection with Ebola virus (EBOV) (*11*,*12*), Marburg virus (*13*), and Rift Valley fever virus (RVFV) (*14*–*16*). Recently, the implications of viral persistence in the eye and other immunoprivileged sites have been highlighted in Ebola virus disease (EVD) (*12*,*17*). The possibility of LASV persistence in the eye is unknown, as is the extent of chronic pathologic changes secondary to infection that could result in long-term functional abnormalities.

Inbred Strain 13 guinea pigs almost uniformly die of disease after LASV infection with the prototypic 1976 Josiah strain without requiring serial adaptation (*18*). In addition, we recently described nonlethal disease in Strain 13 guinea pigs infected with a 2015 isolate from a person with LF imported to New Jersey, USA, from Liberia (LASV 812673-LBR-USA-2015, or LASV-NJ2015 [*19*]). To investigate ocular manifestations of LASV infection in animals that died of or survived infection, we collected samples from animals infected with either LASV-Josiah or LASV-NJ2015. LASV loads and distribution, and associated ocular histopathology, were assessed in these animals.

## Material and Methods

### Ethics Statement

All animal procedures were approved by the Centers for Disease Control and Prevention (CDC; Atlanta, GA, USA) Institutional Animal Care and Use Committee (IACUC; #2833SPEGUIC) and conducted in accordance with the Guide for the Care and Use of Laboratory Animals (*20*). CDC is fully accredited by the Association for Assessment and Accreditation of Laboratory Animal Care International. Procedures conducted with LASV or LASV-infected animals were performed in the CDC Biosafety Level 4 laboratory.

### Virus

Recombinant LASV-Josiah, based on the sequence of an isolate obtained in 1976 from the serum of a 40-year-old man hospitalized at Songo Hospital in Sebgwena, Sierra Leone (*21*,*22*), was rescued in BSR-T7/5 cells and passaged twice in Vero-E6 cells (GenBank accession nos. HQ688673.1, HQ688675.1). Recombinant LASV 812673-LBR-USA-2015 (LASV-NJ2015), based on the sequence of an isolate obtained in 2015 from a 55-year-old man who died of LF in New Jersey after returning from Liberia, was rescued in BSR-T7/5 cells and passaged twice in Vero-E6 cells (*19*) (GenBank accession nos. MG 812650, MG812651). We determined focus-forming units and 50% tissue culture infectious dose (TCID_50_) titers in Vero-E6 cells by immunofluorescence assays using an in-house anti-LASV monoclonal antibody mix targeting nucleoprotein and glycoprotein 2 (SPR628), with TCID_50_ titers calculated using the method of Reed and Muench (*23*).

### Guinea Pig Infections

Sixteen strain 13/N guinea pigs (8 male, 8 female, 6 months to >3 years of age) were obtained from our breeding colony at CDC. Age- and sex-matched 13/N guinea pigs were inoculated subcutaneously with 10^4^ focus-forming units (equivalent to ≈2 × 10^4^ TCID_50_) of LASV, either LASV-Josiah (10 animals) or LASV-NJ2015 (6 animals). Four animals infected with LASV-Josiah served as unvaccinated controls in parallel studies (*24*). All animals were housed individually and given daily fresh vegetable enrichment, hay, commercial guinea pig chow, and water as desired. Experienced CDC veterinarians or animal health technicians assessed animal health. Animals were humanely euthanized with isoflurane vapors and sodium pentobarbital (SomnaSol Euthanasia-III solution; Henry Schein Animal Health, https://www.henryscheinvet.com) once clinical illness scores (including piloerection, ocular discharge, weight loss >25%, changes in mentation, ataxia, dehydration, dyspnea, or hypothermia) indicated the animal was in the terminal stages of disease, or at the completion of study 41 days postinfection (dpi).

### Quantitative Reverse Transcription PCR

RNA was extracted from blood and homogenized tissue samples using the MagMAX-96 Total RNA Isolation Kit (Thermo Fisher Scientific, https://www.thermofisher.com) on a 96-well ABI MagMAX extraction platform with a DNase-I treatment step, according to the manufacturer’s instructions. RNA was quantified by a quantitative reverse transcription PCR (qRT-PCR) targeting a strain-specific nucleoprotein gene sequence (primer and probe sequences available on request from the authors), and normalized to 18S RNA levels. We determined viral small (S) segment copy numbers using standards prepared from in vitro–transcribed S segment RNA.

### Histochemical Staining and Immunohistochemical Analysis

Tissue specimens were fixed in 10% neutral buffered formalin and subjected to gamma irradiation (2 × 10^6^ rad). Formalin-fixed tissues from all guinea pigs were routinely processed, embedded in paraffin, sectioned at 4 μm, and stained with hematoxylin and eosin. A veterinary pathologist visually assessed inflammation within the eye as minimal (few scattered lymphocytes around vessels), mild (small clusters of lymphocytes around vessels or within the filtration angle), or moderate (noticeably more intense infiltrates of lymphocytes within the eye). A marked response, which we did not observe in these animals, would have comprised tissue architecture disrupted by inflammatory cells.

We conducted immunohistochemical (IHC) assays using indirect immunoalkaline phosphatase detection on 4-μm sections. Colorimetric detection of attached antibodies was performed using the Mach 4 AP polymer kit (Biocare Medical, https://biocare.net) at room temperature, except for heat-induced epitope retrieval. Using either Reveal or EDTA buffer, we conducted heat-induced epitope retrieval using the NxGen decloaker (Biocare Medical) at 110°C for 15 min. All slides were blocked in Background Punisher (Biocare Medical) for 10 min and incubated with primary antibody for 30 min. Antibodies used were anti-CD3 (diluted 1:100 in EDTA buffer [#NCL-L-CD3–565; Leica Biosystems, https://www.leicabiosystems.com), anti-CD79a (1:100, EDTA buffer [#NCL-L-CD79a-22; Leica Biosystems]), and a mouse monoclonal antibody targeting LASV glycoprotein 2 at 1:1,000 (CDC). Mach 4 Probe was applied for 10 min, followed by Mach 4 AP polymer for 15 min (Biocare Medical). The antibody/polymer conjugate was visualized by applying Fast Red Chromogen dissolved in Naphthol Phosphate substrate buffer to tissue sections for 20 min (Thermo Fisher Scientific). Appropriate negative control serum was run in parallel. Slides were counterstained with Mayer’s hematoxylin (Poly Scientific, https://www.polyrnd.com) and blued in lithium carbonate (Poly Scientific). Positive controls included formalin-fixed, paraffin-embedded Vero-E6 cells infected with LASV, tissue from a human with LF, and guinea pig spleen and liver (for inflammatory cell and cell lineage markers). A veterinary pathologist scored IHC staining on a scale of 0 (no IHC staining seen) to 4 (abundant, intense IHC staining within structures in the eye).

We performed double staining of antigens after heat-induced epitope retrieval using the EnVision G|2 Doublestain System Rabbit/Mouse (DAB+/Permanent Red; Agilent, https://www.agilent.com) according to the manufacturer’s instructions. We incubated slides in Endogenous Enzyme block for 5 min, primary antibody for 30 min, horseradish peroxidase-polymer for 10 min, and diaminobenzidine (DAB) working solution for 10 min. Double-stain block was then applied for 3 min, and the second stain procedure consisted of applying the other primary antibody for 30 min, followed by addition of the Rabbit/Mouse Link, AP-Polymer, and Permanent Red working solution for 10 min each. Slides were double-stained with the LASV monoclonal antibody (labeled in Permanent Red) and platelet endothelial cell adhesion molecule (PECAM; 1:10 dilution in EDTA buffer [#MU241-UC; BioGenex, https://www.biogenex.com/; labeled with DAB). Appropriate negative control serum was run in parallel. Slides were counterstained, and coverslips were applied.

## Results

### Detection of LASV in Eyes of Animals with Terminal Disease but Not in Survivors

All animals from which samples were obtained demonstrated >1 clinical signs of LASV infection, including elevated body temperature, weight loss, hunched posture, ruffled fur, and altered mentation. Swollen, red conjunctiva with associated ocular discharge was observed in most of the animals, coinciding with onset of clinical signs. To determine whether LASV infects the eye, we collected 1 eye from each of 16 guinea pigs (10 infected with LASV-Josiah and 6 infected with LASV-NJ2015) for PCR to detect viral nucleic acids. The other eye from each animal was used to create full ocular sections that were IHC stained to assess presence and localization of viral antigens.

We detected viral nucleic acids in the eyes of all guinea pigs that died of infection (9 of 10 guinea pigs infected with LASV-Josiah), ranging from 1.32 × 10^5^ to 6.71 × 10^6^ S-segment copies per μL of eluted RNA. Three of the 7 surviving animals, 1 infected with LASV-Josiah (Jos-2) and 2 infected with LASV-NJ2015, had detectable viral RNA within the eye, ranging from 2.50 × 10^1^ to 4.28 × 10^2^ copies per μL of eluted RNA. Viral RNA was below the limit of detection in the remaining survivors (4 guinea pigs infected with LASV-NJ2015).

**Figure 1:**
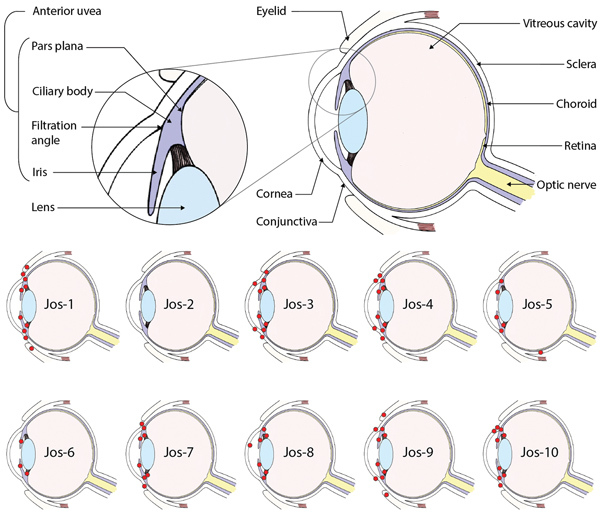
Lassa virus (LASV) localization in guinea pigs that died of or survived infection with LASV-Josiah in study of LASV targeting of anterior uvea and endothelium of cornea and conjunctiva in eye. Primary diagram at top shows major structures of the eye; smaller diagrams detail the general regions in which LASV antigen (red circles) was detected in the eye of each animal by immunohistochemical analysis. All animals were euthanized because of disease (14–23 days postinfection) except Jos-2, which did not exhibit overt clinical signs (no weight loss or elevated body temperature) and was euthanized at study completion (41 days postinfection).

**Figure 2:**
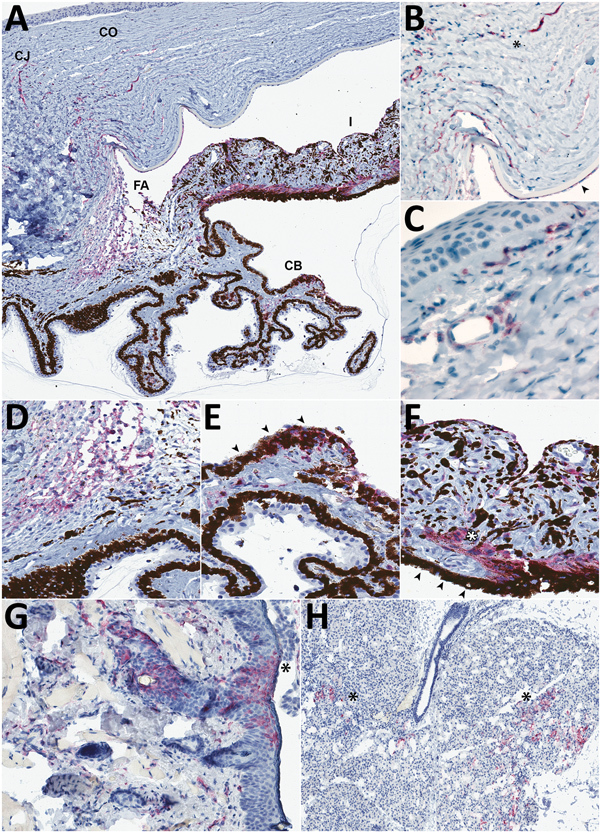
Detection of Lassa virus (LASV) antigen in the anterior uvea and endothelium within the eye of guinea pigs infected with LASV-Josiah and in the epithelium of structures adjacent to the eye in study of LASV targeting of anterior uvea and endothelium of cornea and conjunctiva in eye. A) Anterior uvea with LASV antigen immunolabeled (red) within the peripheral CO and CJ vessels, the FA, CB, and I. Original magnification ×4. B) Immunohistochemical (IHC) staining in the endothelium and adjacent stroma of the corneal margin (asterisk) and in the endothelium deep to Descemet’s membrane (arrowhead). Original magnification ×20 with 1.25 Optivar. C) Perivascular and endothelial staining in the bulbar conjunctiva. Original magnification ×63. D) IHC staining in the filtration angle. Original magnification ×20. E) Photomicrograph of the ciliary body highlighting the labeling in the pigmented epithelium (arrowheads) and stroma. Original magnification ×30. F) Photomicrograph of the iris showing IHC staining of LASV antigen in the stroma, smooth muscle (dilator muscle, white asterisk), and posterior pigmented epithelium (arrowheads). Original magnification ×40. G) IHC staining in eyelid epithelium (asterisk) and dermal vessels in the eyelid. Representative animal Jos-9. Original magnification ×15. H) IHC staining for LASV antigen in the acini of the lacrimal gland (asterisks). Representative animal Jos-9. Original magnification ×5. Representative animals: A–F, Jos-4; F, G, Jos-9. CB, ciliary body; CJ, conjunctival; CO, corneal; FA, filtration angle; I, iris; IHC, immunohistochemical.

We saw no IHC staining in the eyes of any animals that survived infection and detected little to no viral RNA within the eye, including in 1 animal infected with LASV-Josiah (Jos-2) and all animals infected with LASV-NJ2015 (infection confirmed by serology [*19*]). In contrast, IHC staining revealed LASV antigen in the eyes of all animals that died of infection and had detectable viral RNA in the range of 10^5^–10^6^ viral copies. LASV antigen staining was primarily concentrated within anterior regions of the eye (Table; Figures 1, 2). Specifically, we saw staining in the anterior uvea, mostly in the trabecular meshwork at the filtration angle (in 9/9 animals; Figure 2, panels A, D); in the iris, particularly in the pigmented epithelium along the posterior margin, as well as along the anterior margin (in 9/9 animals; Figure 2, panels A, F); and multifocally in the ciliary body epithelium (in 7/9 animals; Figure 2, panels A, E). In addition, we observed perivascular and endothelial staining within the sclera (5/9 animals) and bulbar conjunctiva (5/9 animals) and occasionally in new vessels forming at the peripheral cornea (5/9 animals) (Figure 2, panels A–C). In 5 animals, we noted patchy IHC staining in the corneal endothelium deep to Descemet’s membrane. In 2 animals (Jos-1 and -9), patchy but strong LASV staining was observed in epithelial cells in the surface epithelium of the eyelid, as well as within the dermal vessels, and within acini of the lacrimal glands (Figure 2, panels G, H). In animal Jos-5, we saw staining around scleral vessels at the midline of the eye, in association with mild inflammation.

**Table:** Summary of ocular LASV staining and histopathologic findings in LASV-infected guinea pigs in study of LASV targeting of anterior uvea and endothelium of cornea and conjunctiva in eye*

Guinea pig	**Age at D0/sex**	**dpi**	**Viral RNA†**	**IHC distribution**	**IHC score**	**H&E**
Jos-1‡	3 y 10 mo/F	14	4.55 × 10^5^	Conjunctival endothelium and anterior uvea; eyelid epithelium and endothelium	+++/ ++++	Mild conjunctivitis and anterior uveitis
Jos-2	3 y 10 mo/M	41	2.50 × 10^1^	None	−	Mild perivascular mononuclear inflammation, sclera
Jos-3‡	2 y 5 mo/F	20	1.32 × 10^5^	Anterior uvea, peripheral corneal endothelium	++	Neovascularization at corneal margin, mild conjunctivitis
Jos-4‡	2 y 4 mo/F	17	7.87 × 10^5^	Endothelium, conjunctiva, and peripheral cornea; anterior uvea	+++	Neovascularization at corneal margin, mild conjunctivitis
Jos-5‡	0 y 6 mo/M	17	1.02 × 10^6^	Anterior uvea	++	Mild anterior uveitis
Jos-6‡	0 y 6 mo/M	18	5.32 × 10^5^	Anterior uvea	+	Mild neovascularization at corneal margin
Jos-7‡	3 y 7 mo/F	23	5.23 × 10^6^	Conjunctival endothelium and anterior uvea	++	Neovascularization at corneal margin, mild conjunctivitis, anterior uveitis
Jos-8‡	3 y 3 mo/F	21	2.02 × 10^6^	Anterior uvea, peripheral corneal endothelium	++	Neovascularization at corneal margin, anterior uveitis
Jos-9‡	3 y 6 mo/M	20	6.71 × 10^6^	Conjunctival and peripheral corneal endothelium and anterior uvea, eyelid and lacrimal gland	+++	Neovascularization at corneal margin, moderate conjunctivitis, anterior uveitis
Jos-10‡	>2 y/M	23	2.26 × 10^6^	Conjunctival and peripheral corneal endothelium and anterior uvea	++	Mild neovascularization at corneal margin, minimal anterior uveitis
NJ2015-1	3 y 11 mo/F	41	BLD	None	−	Minimal heterophilic infiltrate at corneal margin
NJ2015-2	3 y 8 mo/M	41	BLD	None	−	NSF
NJ2015-3	2 y 5 mo/F	41	BLD	None	−	NSF
NJ2015-4	2 y 4 mo/F	41	BLD	None	−	NSF
NJ2015-5	0 y 6 mo/M	41	1.54 × 10^2^	None	−	Minimal heterophilic infiltrate at corneal margin, minimal chronic anterior uveitis
NJ2015-6	0 y 6 mo/M	41	4.28 × 10^2^	None	−	Mild anterior uveitis

*All survivors were euthanized at end of study (41 dpi). BLD, below limit of detection (<5 copies); D0, day 0; dpi, days postinfection; H&E, hematoxylin and eosin staining; IHC, immunohistochemical; Jos, guinea pig infected with recombinant LASV-Josiah; LASV, Lassa virus; NJ2015, guinea pig infected with recombinant LASV 812673-LBR-USA-2015; NSF, no significant findings; −, negative results at staining; +, mild immunostaining; ++, moderate immunostaining; +++/++++, abundant immunostaining. †LASV small segment copies in the eye quantified per millileter of eluted RNA. ‡Guinea pig euthanized because of disease.

### LASV Infection in Endothelial Cells in the Eye

**Figure 3:**
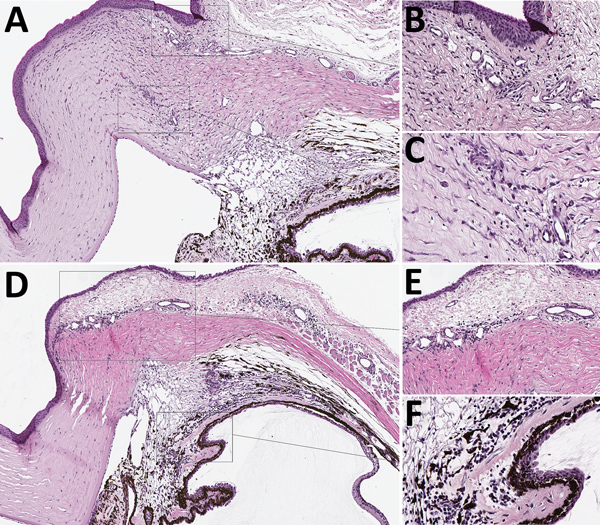
Mild mononuclear anterior uveitis in eyes of guinea pigs infected with Lassa virus (LASV) Josiah by hematoxylin and eosin stain in study of LASV targeting of anterior uvea and endothelium of cornea and conjunctiva in eye. A) Anterior uvea, conjunctiva, and cornea highlighting the mild inflammation and new vessel formation in the peripheral cornea. Original magnification ×4. B) New vessel formation of the peripheral cornea. Original magnification ×12. C) New vessel formation within the cornea highlighting the endothelial swelling and mixed inflammation. Original magnification ×20. D) The ciliary body, filtration angle, peripheral cornea, and a portion of the conjunctiva with mixed, mild, primarily lymphocytic inflammation in the filtration angle and around vessels in the conjunctiva, peripheral cornea, and sclera. Representative animal Jos-1. Original magnification ×6. E) Inflammation around conjunctival vessels at the margin of the cornea. Original magnification ×20. F) Mononuclear inflammation in the filtration angle and at the base of the ciliary body. Original magnification ×20. Representative animals: A–C, Jos-3; D–F, Jos-1.

The distribution of IHC staining in animals with severe disease indicated a predilection for LASV infection of endothelial cells (in the cornea, sclera, conjunctiva, and deep to Descemet’s membrane) and in cells of neural crest and mesenchymal origin (in the iris, ciliary body, and filtration angle [*25*]); we noted viral antigen in endothelial cells and perivascular connective tissues in the conjunctiva and sclera of 5 of 9 animals with terminal disease. In 8 of the 9 animals, we observed mild new vessel formation at the peripheral cornea, with minimal associated inflammation (Figure 2, panel B; Figure 3, panels A–C); LASV staining was noted in endothelial cells of the new vessels in 5 of these guinea pigs (Jos-3, Jos-4, Jos-8, Jos-9, and Jos-10; Figure 2, panels A, B).

**Figure 4:**
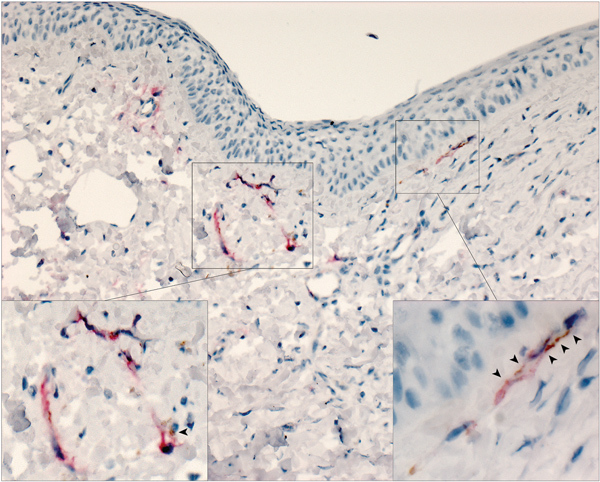
Lassa virus (LASV) targeting endothelial cells in the eye in study of LASV targeting of anterior uvea and endothelium of cornea and conjunctiva in eye. Co-staining for LASV antigen (red) and platelet endothelial cell adhesion molecule (an endothelial marker, brown) in vessels at the margin of the cornea and within the conjunctiva show co-localization of LASV and endothelial antigens (arrowheads). Original magnification ×10; insets enlarged to ×63.

To confirm that LASV targets endothelial cells within the eye, we conducted IHC costaining with an anti-PECAM (anti-CD31) antibody and the anti-LASV antibody. In 50% of animals tested (3/6), we observed viral antigen and PECAM co-staining within vessels in the bulbar conjunctiva and in the newly formed vessels in the peripheral cornea (Figure 4).

### Lymphocytic Ocular Inflammation Caused by LASV Infection

On histopathologic investigation, 56% (5/9) of animals that reached endpoint criteria had mild mononuclear anterior uveitis, with perivascular inflammation composed of lymphocytes primarily in the pars plana of the ciliary body, located near the iridoscleral junction, and scattered within the margins of the trabecular meshwork of the filtration angle (representative animal Jos-1; Table; Figure 3, panels D, H). Inflammation rarely extended into the stroma of the iris. Another prominent feature in 6 of 9 animals that died of LASV infection was mild to moderate lymphocytic inflammation around vessels in the bulbar conjunctiva and the anterior sclera, especially at the corneoscleral junction, and adjacent to the filtration angle (Figure 3, panels D, E). Mild conjunctival hemorrhage was noted in these animals histologically. Almost all (8/9) animals with terminal disease had mild, mixed inflammation composed of heterophils and lymphocytes at the peripheral cornea, as well as mild peripheral corneal neovascularization (representative animal Jos-4; Figure 3, panels A–C); 4 of these animals had a small amount of associated necrotic nuclear debris (Figure 3, panel C). The lymphocytic inflammation in the anterior uvea was most prominent in Jos-1, the guinea pig that had the most acute clinical course and died 14 dpi.

**Figure 5:**
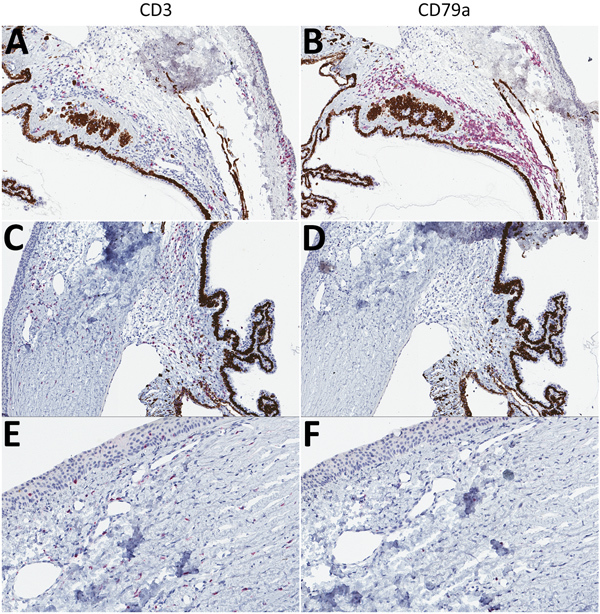
T-lymphocyte inflammation predominant in the eyes of animals that died of Lassa virus (LASV) infection >17 days postinfection in study of LASV targeting of anterior uvea and endothelium of cornea and conjunctiva in eye. CD3+ (left) and CD79a+ (right) lymphocyte antigens targeted by immunohistochemical (IHC) analysis are stained red. A) Inflamed filtration angle and sclera highlighting CD3+ T-lymphocytes. Original magnification ×10. B) Inflamed filtration angle and sclera highlighting the predominant population of CD79a+ B-lymphocytes. Original magnification ×10. C) Mildly inflamed filtration angle and sclera showing the predominance of CD3+ T-lymphocytes within the region. Original magnification ×10. D) Absence of CD79a+ B-lymphocytes. Original magnification ×10. E) New vessel formation at the margin of the cornea, indicating scattered CD3+ T-lymphocytes. Original magnification ×20. F) Minimal CD79a+ B-lymphocytes. Original magnification ×20. Representative animals: A, B, Jos-1; C–F, Jos-5.

To characterize the inflammatory cell populations in animals with terminal disease, we conducted IHC staining targeting CD3+ T-lymphocytes and CD79a+ B-lymphocytes. The composition of inflammatory cells varied based on disease duration. The inflammatory milieu comprised T-lymphocytes and B-lymphocytes in Jos-1, which died of disease 14 dpi (Figure 5, panels A, B), but consisted primarily of T-lymphocytes in all animals that died >17 dpi (representative animal Jos-5; Figure 5, panels C–F). T-lymphocytes were most abundant in the trabecular meshwork and in the perivascular connective tissue in the sclera and bulbar conjunctiva. Scattered T-lymphocytes also were seen in the peripheral corneal stroma and epithelium in animals with corneal neovascularization (Figure 5, panel E).

Despite the absence of detectable viral antigen, we observed inflammation in all animals infected with LASV-NJ2015, but not in Jos-2, which survived LASV-Josiah infection. However, the degree of inflammation in surviving animals was notably less than in animals with terminal disease. Survivors demonstrated minimal to mild lymphocytic inflammation in the eye, compared with mild to moderate inflammation in animals with terminal disease. The lymphocytic inflammation in the eyes of surviving animals was accompanied by minimal heterophilic infiltrate around vessels of the peripheral cornea in 2 animals (NJ2015-1 and -5; Table). In addition, mild lymphocytic anterior uveitis was seen in NJ2015-6, which had the highest viral RNA copy number among surviving animals.

## Discussion

Long-term ocular manifestations are not well described in LASV infection, but given the importance and persistence of ocular lesions after infection with other hemorrhagic fever viruses, the pathogenesis of LASV in immune-privileged sites such as the eye must be fully characterized. In the few reported cases of ocular involvement in acute LF, clinical findings have primarily been described as conjunctivitis and conjunctival edema (*9*), although uveitis resulted in transient blindness in 1 case (*3*). In this study, we observed conjunctivitis and conjunctival edema clinically in infected guinea pigs, as described in human patients with acute disease. LASV RNA was detected by PCR in the eye, and LASV antigen was detected by IHC within the anterior uvea of animals that died of infection, particularly in the endothelium and perivascular stromal cells, and occasionally within epithelium. Similar LASV immunostaining was previously noted in other tissues in this animal model, as were the histologic features of mild lymphocytic inflammation and rare cell death (*26*).

Other animal models of LF also demonstrate infection in the eye. For example, the aqueous humor of the anterior chamber of the eye was found to be heavily infected in rhesus macaques that died of experimental LASV infection (*27*). Perivascular infiltrates of plasma cells and lymphocytes were described in the choroid, sclera, iris, filtration angle, and ciliary body of most of the animals, similar to what we observed here in guinea pigs.

In contrast to the findings in humans with LF and in animal models of LF, ocular manifestations during infection with several other hemorrhagic fever viruses, including filoviruses and phenuiviruses, include a broader tissue tropism in the eye and some viral persistence within the eyes of survivors. During the most recent EVD outbreak, infectious virus was detected months after clinical resolution in the intraocular aqueous humor of 1 person (*11*), and uveitis was reported in 18% of a group of EVD survivors in Sierra Leone (*12*). Uveitis, both anterior and posterior, was described in a series of survivors of the 1995 EVD epidemic in the Democratic Republic of the Congo (*12*,*28*). Anterior uveitis was also described after infection with the related Marburg virus, and the virus was subsequently cultured from the aqueous fluid of this patient (*13*). Macular, paramacular, or extramacular retinal lesions, with hemorrhage, edema, vasculitis, and retinitis, often occurring bilaterally, have been reported in association with RVFV infection. Patients were monitored during a 6-month convalescence after RVFV infection, and though lesions were resorbed, approximately half of the patients permanently lost visual acuity (*15*,*16*). Subsequent studies from an RVFV outbreak in Saudi Arabia in 2000 reported similar findings (*14*).

Recent studies have focused heavily on ocular involvement in EVD, resulting in detailed clinical descriptions in patients and prioritization for evaluation in animal models. In primates with an acute course of disease that resulted in death, EBOV RNA was not detected in the parenchymal ocular tissues but was consistently detected in the blood vessels of the choroid or ciliary processes or in the optic nerve leptomeninges (*29*). In this nonhuman primate EVD model, EBOV persisted in the vitreous humor, in cells attached to the retinal inner limiting membranes, and in the ciliary body, with a predilection for CD68+ macrophage/monocytes, and with associated uveitis, retinitis, and vitritis (*29*). In contrast, we found that guinea pigs that died of LASV infection had viral antigen only in anterior regions of the eye, whereas surviving animals did not have LASV antigen and only showed minimal inflammation within the eye.

One major site of viral localization in this study was within endothelial cells in the conjunctiva and peripheral cornea. This finding correlated with studies describing the ability of LASV to replicate to high levels in endothelial cells and alter cytokine expression in cell culture (*30*). Another feature of ocular LASV infection noted in this study was the associated chronic inflammation, composed primarily of T-lymphocytes, in the anterior uvea, conjunctiva, and cornea in animals that died at later time points in the infection (>17 dpi). Lymphocytic anterior uveitis has been described in ocular infections with other viruses (e.g., rubella virus, cytomegalovirus, herpes simplex virus, and chikungunya virus) and can indicate a secondary immune response to viral antigens (*31*,*32*), although the pyknotic debris and swollen endothelium in the new vessels at the corneal margin in LASV-infected guinea pigs suggest a more acute insult. A predominantly T-lymphocyte response has been documented as a particularly important component of the systemic immune response to LASV infection (*33*).

Our studies in the strain 13/N guinea pig model indicate that LASV is present in the eye and elicits an inflammatory response primarily during acute clinical disease that is only minimally detected in convalescence. These features echo clinical findings in LF survivors, in whom ocular disease has not been described. The lack of reported ocular disease in LF survivors, along with the presence of only minimal or mild pathology in surviving animals, suggests less frequent or less severe ocular sequelae of LF than described in other viral hemorrhagic fever diseases. However, because similar mild inflammation has been associated with iris atrophy and ocular hypertension in other viral infections (*31*,*32*), the low degree of inflammation seen in our study is not necessarily innocuous. Although these data and the few clinical reports of ocular involvement in survivors of human LF disease support minimal long-term effects on vision, careful ophthalmologic observation of LF survivors is warranted, along with further longitudinal studies in the subpopulation of animal models that survive LASV infection despite clinical signs. These studies would aid in determining whether the presence of LASV and the resultant inflammation, even after clearance, produce long-term sequelae in the eye.
